# Nail Salon a Potential Source of a Rare Mycobacterium Fortuitum Infection In Proximal Tibia Megaprosthesis? A Case Report

**DOI:** 10.7150/jbji.43023

**Published:** 2020-04-27

**Authors:** Thomas A. Novack, Tyler Hoskins, Jay N. Patel, Christopher Mazzei, David Goyette, Kaitlin Zeedyk, James C. Wittig

**Affiliations:** 1 Department of Orthopedic Surgery, Morristown Medical Center, Morristown, NJ; 2 Department of Orthopedic Surgery, St. Joseph's Regional Medical Center, Paterson, NJ

**Keywords:** Infection, Mycobacterium Fortuitum, Parosteal Osteosarcoma

## Abstract

Mycobacterium Fortuitum (M. Fortuitum) is a type of opportunistic pathogen commonly found in water/soil and belongs to the nontuberculosis mycobacteria (NTM) family. Prosthetic joint infection due to M. Fortuitum is extremely rare. We present a case of a 21-year-old female with an infection following a radical resection of the proximal tibia due to a parosteal osteosarcoma.

## Introduction

**Figure 1:**
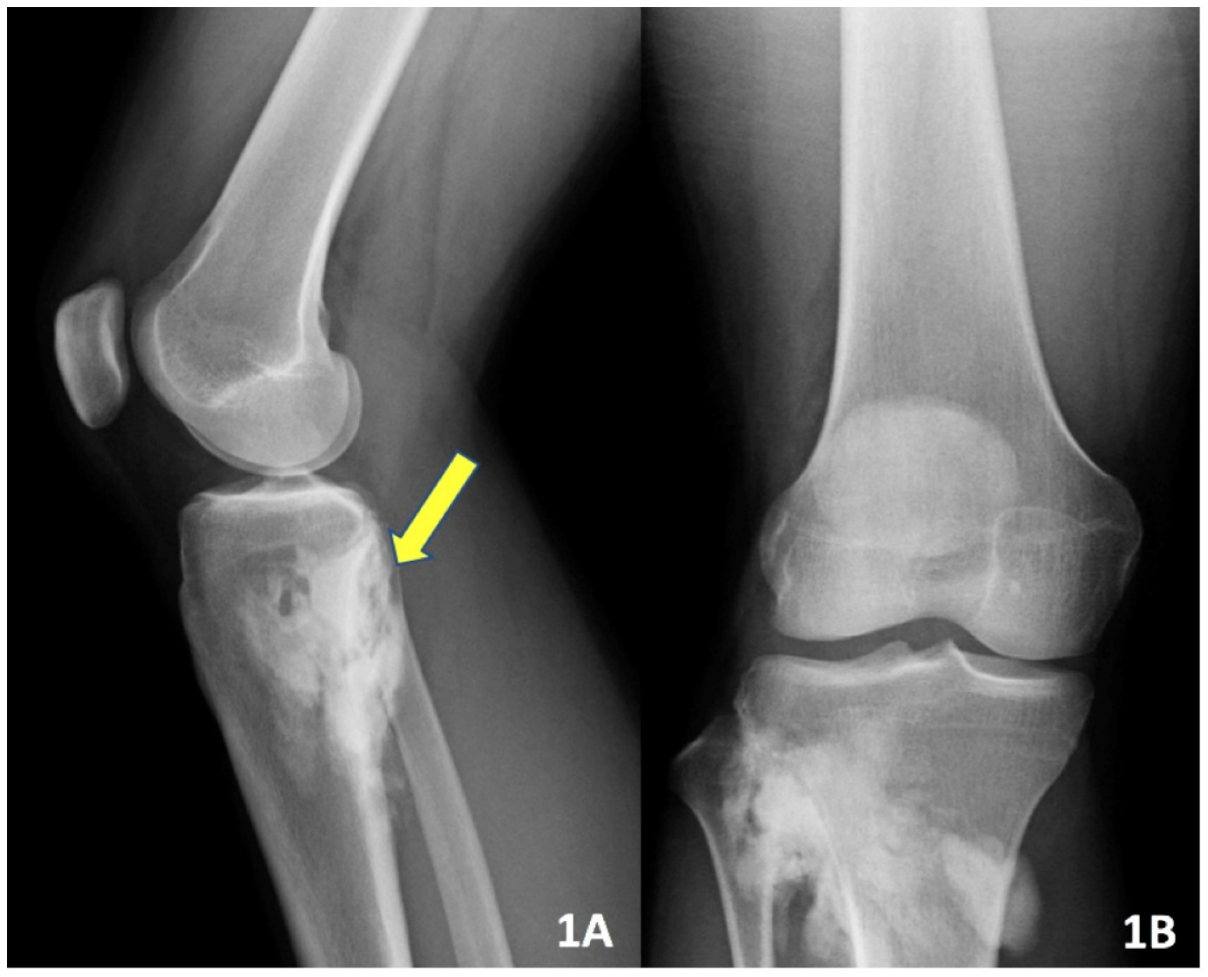
Pre-op lateral (A) and AP (B) x-ray imaging demonstrating an irregular osseous lesion of the right proximal tibia and fibula.

**Figure 2:**
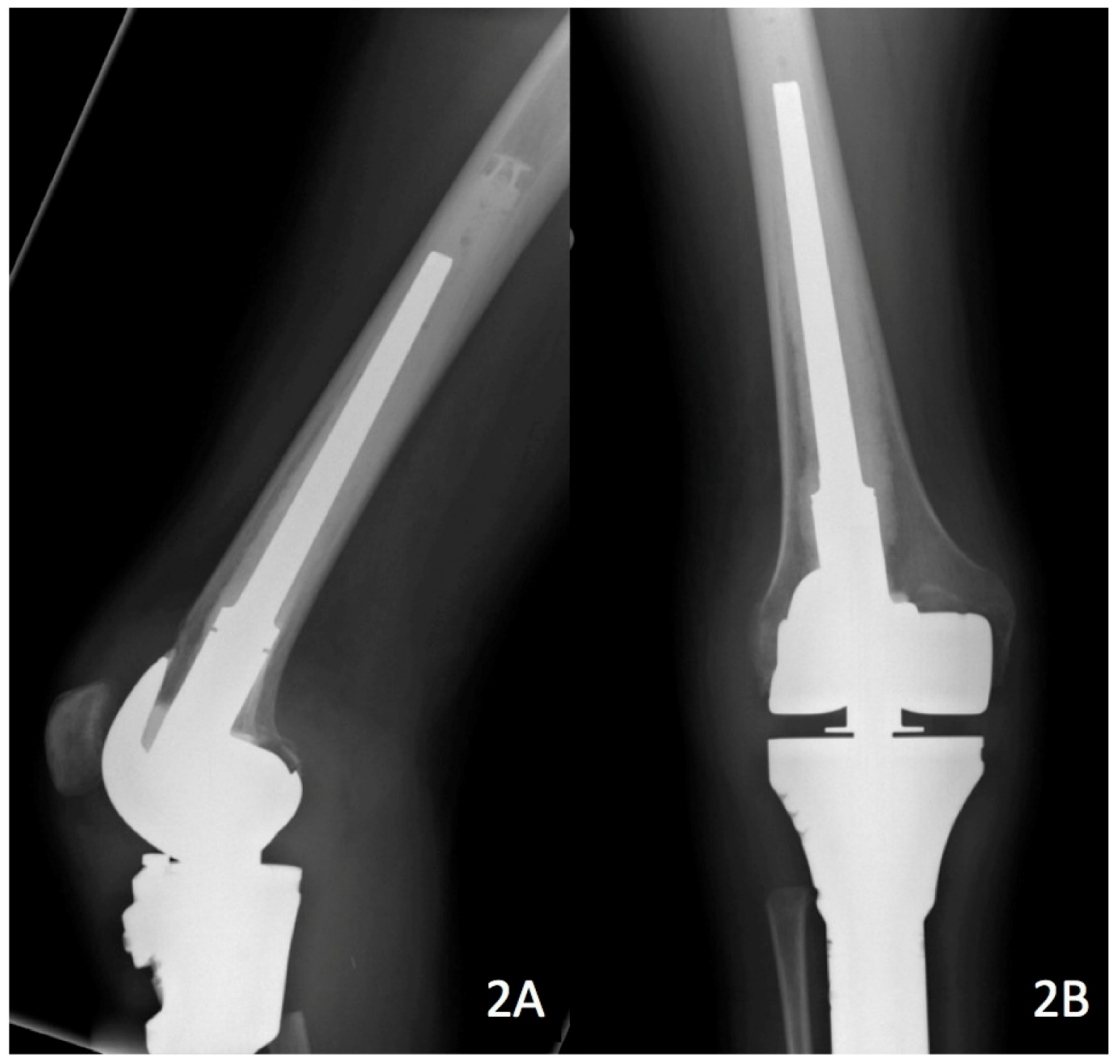
8 week post-op lateral (A) and AP (B) x-ray imaging demonstrating no signs of recurrence, hardware failure, or infection.

A 21-year-old female with a past medical history of irritable bowel syndrome and Lyme disease presented to our office with right knee pain. She was found to have a 9 cm parosteal osteosarcoma of the proximal tibia (Figure 1). She underwent a radical resection and reconstruction with an 11 cm modular segmental tumor prosthesis (Link; Hamburg, Germany) along with a rotational medial gastrocnemius muscle flap. A negative-pressure wound care system was used and the patient was also placed in a posterior leg splint. The incisional wound vacuum was prophylactically used at index procedure to promote faster wound healing and aid it by taking tension off of the incision as well as the gastrocnemius muscle flap. There was no communication between the sponge and the patient's tumor prosthesis. Patient's immediate postoperative course was unremarkable. Thirteen days after surgery the patient's dressings were taken down and the incision was healing well. The patient was then placed in a hinged knee brace locked in extension for a total of 6 weeks after surgery to allow her extensor mechanism to heal. Flexion was gradually permitted starting at week 6. At 8 weeks post-operatively the patient had a well-healed surgical incision and radiographs demonstrated all implants were in excellent position (Figure 2). The patient was instructed to start more progressive range-of-motion exercises with physical therapy.

**Figure 3:**
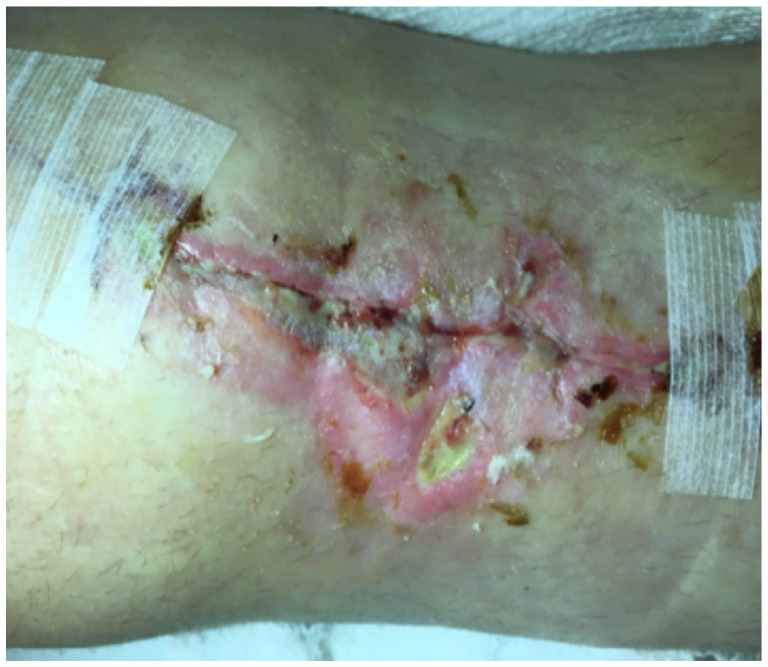
Wound 11 weeks post-op.

At 11 weeks post-operatively, the patient presented with a small cutaneous abscess on the inferior aspect of her incision. The incision became erythematous and began to produce a purulent discharge (Figure 3). At this time, the patient gave a history about going to the nail salon to have a pedicure in the weeks prior. She underwent irrigation and debridement in the operating room. The infection was superficial and did not go deep down to the implant. Cultures were sent off during surgery for aerobic, anaerobic, acid fast bacilli (AFB), and fungal. In our orthopedic oncology practice, many patients are immunocompromised secondary to chemotherapy and so we routinely send specimens for aerobic, anaerobic, AFB, and fungal cultures when infection is suspected. Initial gram stain, aerobic, and anaerobic cultures were negative and the patient was discharged with a PICC line on empiric daptomycin and piperacillin/tazobactam. Twenty-one days after the debridement procedure, the acid-fast bacillus cultures became positive for *M. fortuitum* (Table 1).

At this time the patient was readmitted to the hospital and underwent repeat irrigation and debridement of the surgical wound due to continued erythema and drainage to also include the deeper tumor prosthesis in an attempt to preserve the initial prosthesis. She was started on intravenous amikacin 750mg daily and imipenim/cilastin 500mg every 6 hours as well as oral ciprofloxacin 750mg twice daily. The patient was seen 4 and 8 weeks after the second debridement procedure with minimal improvement in her inflammatory markers and clinical picture.

The patient was subsequently admitted to the hospital and, approximately 6 months after the index procedure, underwent removal of the tumor megaprosthesis and placement of a static antibiotic spacer; the tibial and femoral stems were left in place and the wound and surrounding tissues were copiously scrubbed with chlorhexidine, hydrogen peroxide, and betadine. Post-operatively she was started on intravenous imipenim/cilastin 500mg every 6 hours, oral ciprofloxacin 750mg twice daily, and oral minocycline 100mg twice daily. 8 weeks later, after the patient had completed her antibiotics and her laboratory values had normalized, she underwent exchange of the antibiotic spacer for a new megaprosthesis. Post-operatively, she was re-started on amikacin 750mg IM three days a week, intravenous imipenim/cilastin 500mg every 6 hours, and oral ciprofloxacin 750mg twice daily until all of her intraoperative cultures were finalized. All operating room cultures at the time of exchange, including aerobic, anaerobic, fungal, and AFB were negative. On subsequent post-operative visits the patient demonstrated excellent healing and after completing her initial course of post-operative antibiotics she was transitioned to oral doxycycline 100mg twice daily for one year given her history of multiple surgeries, cancer diagnosis, and her infection. At 24-months post-revision surgery she is doing well with the implants in good position with no evidence of infection.

## Discussion

NTM are an atypical type of mycobacteria that do not cause tuberculosis or leprosy. M. Fortuitum is a member of the rapidly growing Reunion Group IV NTM and is most frequently acquired from water, soil, and nosocomial sources. It is recognized as an opportunistic pathogen, mainly causing infection and morbidity in immunocompromised patients. However, there have also been cases reported of a M. Fortuitum infection in immunocompetent hosts. Other authors have previously presented prosthetic joint infections (PJI) in primary joint arthroplasty patients by NTM, including *M. fortuitum* 1, 2, 3, 4, 5*.* In many of the cases reported in the literature on M. Fortuitum infections, the standard of treatment was surgical removal of the entire prosthesis, placement of an antibiotic spacer with concomitant intravenous antibiotics, and then replantation after the infection had cleared 1, 2, 3, 4.

However, Eid et al. presented eight patients with nine infections due to rapidly-growing Mycobacteria (seven knees, one hip, one elbow), three of which were *M. fortuitum* 5. Median onset of symptoms was 312 weeks after implantation (range 1-720). Two of these patients, both total knee arthroplasty patients, were treated with debridement and implant retention. The remaining patients all had their implants removed. The two patients with retained prosthesis received treatment with moxifloxacin, trimethoprim-sulfamethoxazole, and azithromycin or levofloxacin and trimethoprim-sulfamethoxazole for long-term suppression of *M. fortuitum* infection. Both were asymptomatic at 24 and 189 weeks after debridement, respectively. In our study, after one superficial and one deep wound debridement with an effort to preserve the initial prosthesis, the infection unfortunately could not be eradicated. The patient was treated with removal of the prosthesis with cemented stem preservation, and insertion of an antibiotic spacer. In addition, the patient was administered IV and oral antibiotics post-operatively. She is currently asymptomatic at 24 months post-operatively. Other authors have looked at factors that may indicate the need to remove prostheses in mycobacterial infections. Harwin et al. found that patients with later onset of symptoms (> 6-8 weeks), presence of a draining sinus, concomitant bacterial infection, osteolysis or prosthesis instability indicate the need to remove the prosthesis 6. In our patient, the decision for stem retention was made because the stems were firmly fixed and removal could result in significant bone loss, thus compromising an ultimate reconstruction. Thus, retention of certain components with appropriate antibiotic treatment may be an option, particularly in major reconstructive cases.

The timing of *M. fortuitum* infections has also been described in the literature. Jitmuang et al. presented a case series of 16 patients with NTM infections, nine of which were *M. fortuitum* 7*.* In their study, they found that of the 11 patients with NTM mycobacterial infection, 9 of the infections occurred within 90 days after the index procedure. This is in contrast with previous literature, which showed more delayed onset of infection 5. In our study, the patient showed the first signs of infection approximately 11 weeks post-operatively. It is also imperative to emphasize that these bacteria may not be readily apparent on gram stains and may require several weeks to grow from AFB cultures. Thus, it is vital to consider an agent such as *M. fortuitum* during any phase in the post-operative period as a potential cause of infection, especially in immunocompromised individuals, patients who have visited a nail salon or been exposed to lake water. The fungal cultures should be followed up for 4-6 weeks after surgery.

One interesting aspect of the case was the patient reporting her recent pedicure at a nail salon after her index procedure. It has previously been reported that there is an association between mycobacterial infections and pedicures, particularly the footbaths performed at many salons 8, 9, 10. Vugia et al. reported an outbreak of mycobacteria in nail salons from 5 California counties. The study team swabbed 30 footbaths in 18 nails salons and found cultures of mycobacteria in 29 (97%) 10. Although it is difficult to draw any direct conclusion from these results, there is still a significant risk factor associated with visiting nail salons, especially for post-operative patients with incisions that may not have fully healed. In addition, while these infections are rare, it is crucial to understand any risk factors for a post-operative patient, regardless of how trivial it may seem. This case was unusual because our patient was immunocompetent, and thus should be less prone to these types of infections. Therefore, when counseling any patient on post-operative care or working up a potential infection, there is a need to know about pedicures as a potential source of infection. Patients undergoing joint replacement surgery should be counseled about the need to refrain from pedicures and manicures, especially in the first 90 days after surgery when the patient is most vulnerable to developing an infection. Surgeons should also be mindful that all post-operative patients are at risk for these rare types of infection, thus every specimen received for pyogenic culture must be processed for AFB examination and culture. Any delay in proper treatment makes it difficult for the surgeon to eradicate this type of bacteria and concomitantly puts the patient at a much higher risk of amputation.

## Conclusion

To our knowledge this is the first case presented of a *M. fortuitum* infection developing post radical resection and reconstruction with a proximal tibia megaprosthesis. This case emphasizes the importance of considering mycobacterium as a source of infection in these patients. Furthermore, it highlights the importance of considering all patient risk factors when determining the appropriate procedure to eradicate the infection. Finally, surgeons may need to consider the risk of post-operative patients receiving a pedicure at a nail salon, however trivial it may seem.

## Author Contributions

All authors contributed to the conception and design of the study. TN, TH, JP, and JW helped write the manuscript. CM, DG, and KZ helped gather information about the patient's case from the hospital database. JW performed all surgeries outlined in this paper.

## Figures and Tables

**Table 1:**
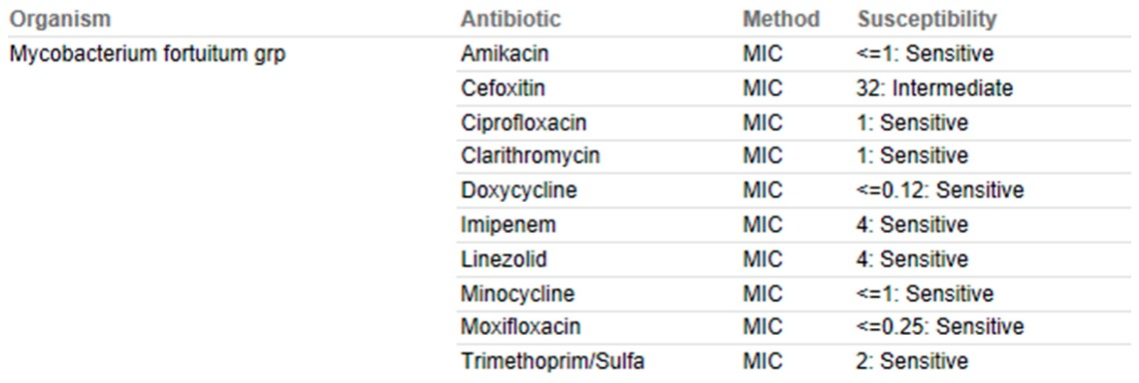
Anti-Mycobacterial Susceptibility Testing und Results
